# Update of the 1972 Singer-Nicolson Fluid-Mosaic Model of Membrane Structure

**DOI:** 10.15190/d.2013.3

**Published:** 2013-12-31

**Authors:** Garth L. Nicolson

**Affiliations:** The Institute for Molecular Medicine, Department of Molecular Pathology, Huntington Beach, CA, USA

**Keywords:** membrane domains, extracellular matrix, lipid rafts, membrane-associated cytoskeleton, membrane asymmetry, membrane dynamics

## Abstract

The Fluid-Mosaic Membrane Model of cell membrane structure was based on thermodynamic principals and the available data on component lateral mobility within the membrane plane [Singer SJ, Nicolson GL. The Fluid Mosaic Model of the structure of cell membranes. Science 1972; 175: 720-731]. After more than forty years the model remains relevant for describing the basic nano-scale structures of a variety of biological membranes. More recent information, however, has shown the importance of specialized membrane domains, such as lipid rafts and protein complexes, in describing the macrostructure and dynamics of biological membranes. In addition, membrane-associated cytoskeletal structures and extracellular matrix also play roles in limiting the mobility and range of motion of membrane components and add new layers of complexity and hierarchy to the original model. An updated Fluid-Mosaic Membrane Model is described, where more emphasis has been placed on the mosaic nature of cellular membranes where protein and lipid components are more crowded and limited in their movements in the membrane plane by lipid-lipid, protein-protein and lipid-protein interactions as well as cell-matrix, cell-cell and cytoskeletal interactions. These interactions are important in restraining membrane components and maintaining the unique mosaic organization of cell membranes into functional, dynamic domains.

## Introduction

The Fluid Mosaic Membrane Model of biological membrane structure was envisioned as a basic framework model for interpreting existing data on membrane proteins and lipids, and their dynamics^1^. At this time, the accepted model for cellular membrane structure was a static tri-layer model of protein-lipid-protein^2^, later refined as the Unit Membrane Model^3^. The tri-layer membrane models were based on the lipid bilayer proposal of Gorter and Grendel^4^, with added unfolded protein beta-sheets on either side of the lipid bilayer and bound to it by electrostatic and other forces. A few trans-membrane proteins were added later (Robertson, 1981)^5^ to reconcile observations on ion transport and freeze-fractured images of cell membranes^6^. Alternatively, lipo-protein Subunit Membrane Models were proposed without a lipid bilayer matrix^7,8^.

**Figure 1:**
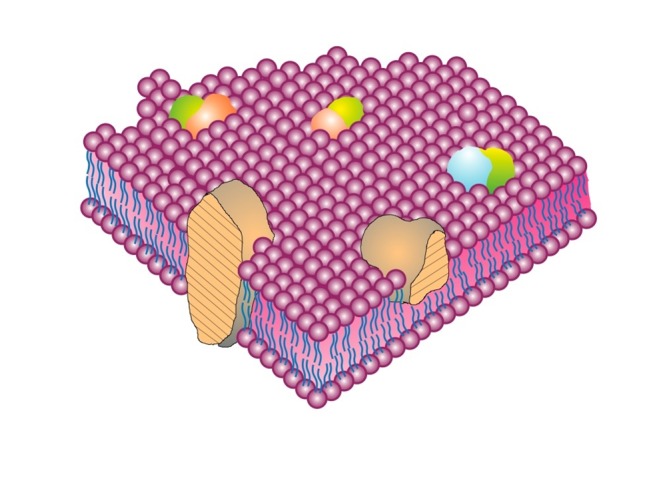
The Singer-Nicolson Fluid- Mosaic Membrane Model of cell membrane structure as proposed in 1972 In this view of a cell membrane the solid bodies with stippled cut surfaces represent globular integral membrane proteins randomly distributed in the plane of the membrane. Some integral membrane proteins form specific integral protein complexes, as shown in the figure. Integral proteins are represented in a fluid lipid bilayer. The model does not contain other membrane-associated structures or membrane domains (from Singer and Nicolson^1^).

The Fluid Mosaic Model^1^was successful because it was based on the natural bilayer formation of membrane glycerophospholipids and the amphipathic structures of integral globular membrane proteins that allowed their intercalation into the hydrophobic lipid bilayer matrix. Only this model considered the different categories of proteins (integral and peripheral) and the ability of components in membranes to rapidly move laterally and change their topographic distributions within a fluid membrane matrix (**Figure 1**)^1^. The bilayer structure of membrane phospholipids and their lateral motions in the membrane plane were consistent with data on membrane structure and have been the subjects of a number of reviews over the years (the early literature can be found in^9-15^). For example, Edidin^12^ reviewed the history of membrane lipid structural proposals over the last century and concluded that cellular membranes must contain a phospholipid bilayer matrix. Also, the proposal that integral or intrinsic proteins exist as globular structures imbedded into the lipid bilayer (in contrast to peripheral or extrinsic membrane proteins that are also present but are not bound to membranes by hydrophobic interactions) remains supported by overwhelming evidence^9-19^.

Although the Fluid Mosaic Model has been remarkably consistent with data collected on biological membranes since the early 1960s^9-21^, it was inevitable that the original model could not explain all of the data in every subsequent study. However, the model quickly evolved from the original 1972 model, and the revisions that occurred within a few years took into account many questions that were raised about the 1972 model^9^. Most of these criticisms came decades after the original model and did not question its fundamental structural principles^19,22-27^.

It is now widely accepted that there are limitations in the Fluid Mosaic Model as originally proposed^1^ in explaining the domain structures present in membranes, especially those membranes found in specialized tissues and cells. Thus, the model has been refined and renamed as the ‘Fluid-Mosaic Membrane Model’ (F-MMM) to highlight the important role of mosaic, aggregate and domain structures in membranes and restraints on the free lateral movements of certain components. Early modifications of the basic F-MMM included the interactions of extracellular matrix and membrane-associated cytoskeletal components with cell membranes and their influences on the mobility and distribution of trans-membrane glycoproteins^9^. Also, the possibility that less mobile lipid-protein or lipid-lipid domains might exist in membranes as frozen or semi-frozen islands in a sea of fluid phospholipids was also proposed^9^. As will be discussed below, the hypothesis that trans-membrane interactions with membrane-associated structures influences membrane dynamics was important in explaining the integration of structure, component mobility, and membrane function^9,10,28^. The relatively recent discoveries of lipid rafts and other specialized membrane domains, membrane-associated ‘fences’ and membrane ‘fenceposts,’ and their possible functions in controlling and restraining membrane protein distribution and mobility continued this trend^10,12,23-37^.

**Figure 2:**
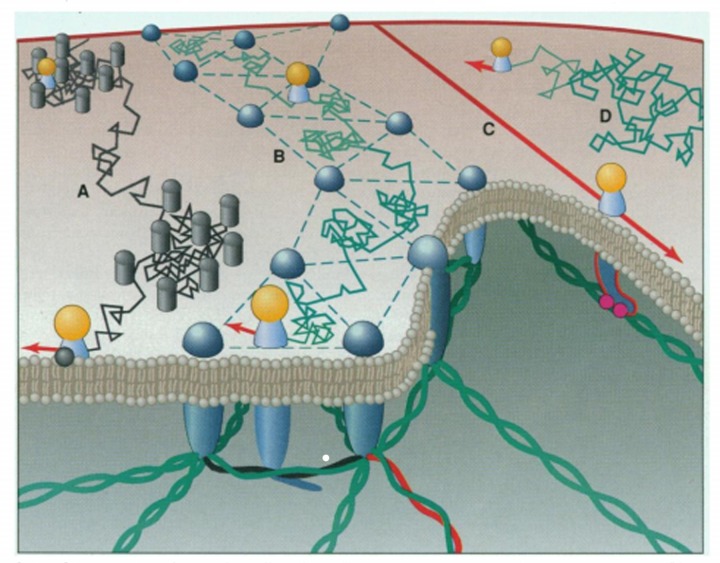
Different examples of cell membrane integral membrane protein lateral mobility as envisioned by Jacobson and colleagues in 1995 Integral membrane protein lateral move-ments are described as: transient confinement by obstacle clusters (**A**); transient confinement by the cytoskeleton (**B**); directed motion by attachment to the cytoskeleton (**C**); and free, random diffusion in the membrane plane (**D**) (from Jacobson et al.^23^).

Models of cell membranes produced a few years after the 1972 model were much less homogeneous looking than the original model^9^. They contained additional information not included in the original model, such as protein and lipid aggregations and segregation into domains, cytoskeletal and matrix interactions, among other features (some of these are shown in Figure 2 of Nicolson^9^). Importantly, revisions of the F-MMM retained all of the basic elements of the original model. In newer models of biological membranes, the arrangement of compo-nents into more compact structures and domains maximizes the mosaic nature of these structures (the historical development of the F-MMM and supporting data from the previous century, are discussed in^10^).

## Physical Principles and Cell Membranes

Singer^14^drew attention to the work of Kauzmann^38^ and the important concept of hydrophobic interactions in maintaining cell membrane structure. Hydrophobic structures self-associate to exclude water interactions, whereas hydrophilic structures interact with the aqueous, ionic environment. Membrane phospholipids self-assemble to form bilayers due to the energy provided by the hydrophobic effect and van der Waals forces^39^. Membrane integral globular proteins interact with membrane lipids, mainly their acyl tails, due to hydrophobic forces and much less to hydrophilic interactions between the lipid head groups and protein hydrophilic groups^15,39,40^. As originally proposed^1,14^, the basic nano-scale structural organization of membranes has remained relatively consistent with current evidence^9,10,15,18,21-24,40-45^, with some modifications^26,27,32,34,35,40,45^. This will be discussed below.

Cell membranes are dynamic structures that are quite susceptible to deformation. For example, membranes deform when confronted with forces less than those driven by the hydrophobic effect^46^. Membrane deformation depends on the energies of lipid tilt and splay, which in turn are dependent on lipid composition^46^. Lipids that support positive spontaneous curvature can reverse the effects of lipids that support negative spontaneous curvature, and this may be important in membrane fusion and other dynamic membrane-membrane interactions^47^.

Different lipids and integral membrane proteins must adjust to each other’s hydrophobic structures. Thus, Israelachvili^40^ proposed that the F-MMM required refinement to account for these differences. Further, Mouritsen and Bloom^48^ suggested that sorting of lipids and proteins was based on the interactions of their hydrophobic regions and to a lesser degree their hydrophilic interactions in order to prevent mismatches between lipids and proteins. This concept was incorporated into the Mattress Model of membranes that described how variations in the hydrophobic parts of lipids and proteins drive associations or hydrophobic matching between different types of membrane components to prevent membrane distortions^48^. This concept will be considered again in another section.

Membrane distortions, such as deformation, curvature, compression and expansion, are driven by different forces and components within membranes^46-51^. For example, certain soluble proteins can bind to membranes and cause deformation. Examples are proteins that contain BAR (Bin/ amphiphysin/ Rvs) domains that form crescent-shaped α-helical bundles that bind to membranes via electrostatic and hydrophobic interactions. BAR proteins can generate membrane curvature by scaffolding to the inner surface of the membrane, causing it to bend to the curvature of the protein^52^. When inserted into a membrane some proteins alter their shape by undergoing folding transitions to form α-helices that wedge between membrane components, thus deforming the membrane and causing membrane curvature^53^.

## Cell Membrane Asymmetry

Biological membranes are asymmetric structures^1,9-11,18,54-56^. The asymmetric nature of cell membranes was actually known well before the original F-MMM was proposed (for example, Stoeckenius and Engelman^56^). One reason for this is that the free energy required to flip membrane amphipathic lipids and proteins across the hydrophobic membrane interior is substantial; thus, cell membrane flip-flop that could result in symmetric structures should be rare^1,13,14^.

Membrane lipid asymmetry is essential in guiding membrane curvature and other aspects of membrane structure^50^. The compositional differences between the inner and outer leaflets of cell membranes suggest that the outer leaflet is curvature neutral, while the inner leaflet may have a preference for negative curvature^46^. The finding of asymmetric distributions of various phospholipids between the inner and outer leaflets of cell membranes has proved to be relatively monotonous^54-60^. For example, the enrichment of amine- and serine-containing phospholipids found on the inner surface and choline-containing phospholipids and spingomyelins on the outer surface of cell membranes creates increased affinity of cholesterol to the outer bilayer leaflet, and this might have some advantage in terms of membrane associations into domains and maintenance of enzymatic activities^12,21,60-63^.

There is a cost for not maintaining appro-priate cell membrane asymmetry, and it is not just the appropriate display of enzymes, receptors and other functional components of membranes. Disruption of the normal membrane asymmetry is associated with cell activation (activation of cell adhesion, aggregation, apoptosis, recognition by phagocytic cells, among other events), and it can also be associated with pathologic processes^58^.

It follows that a number of lipid transporters have been discovered that are important in maintaining lipid asymmetry^57-59^. Examples include the inner membrane-directed, ATP-dependent transporters (‘flippases’), outer membrane-directed, ATP-independent transporters (‘floppases’), and the bidirectional, ATP-indepen-dent transporters (‘scramblases’)^58,59^. The existence of these phospholipid transporters in maintaining the proper phospholipid asymmetries in cellular membranes suggests that maintenance of membrane asymmetry is functionally important.

Membrane integral protein asymmetry is easier to explain, but certainly no less complex^115-17,64^. It is probably initiated at the time of protein synthesis during the initial insertion of the polypeptide chains into the membrane mediated by molecular gatekeepers called translocons^16,17^. Since the energy required to flip integral globular membrane proteins across a hydrophobic barrier is enormous, integral membrane protein asymmetry does not have to be actively maintained after biosynthesis.

## Membrane Proteins and Membrane-Associated Proteins

As discussed in the original publication on the F-MMM^1^, it was important to distinguish between the integral (or intrinsic) proteins that are tightly bound to membranes by mainly hydrophobic forces and intercalated into the membrane hydrophobic matrix and peripheral (or extrinsic) proteins that are loosely bound to membranes by electrostatic or other non-hydrophobic interactions. Numerous examples of both types of membrane proteins abound, and this has been reviewed in more detail elsewhere^14-17^. I have discussed the importance of integral membrane proteins in defining basic membrane nano-scale structure of cell membranes; however, peripheral membrane proteins also have an important role, but not necessarily in maintaining the basic structures of membranes. They appear to be more important in providing non-membrane protein attachment sites, scaffolding, tethering or membrane -supporting structures, membrane curvature-preserving components, and attachment points for soluble enzymes and signaling molecules.

Peripheral (or extrinsic) membrane proteins were originally operationally defined as proteins that could be removed from membranes without destroying basic membrane microstructure^1,13^. This was an operational not an exact definition to help explain the roles of different membrane proteins in defining basic membrane micro-structures. Peripheral membrane proteins do not have to be strictly globular in structure, and certainly not amphipathic, and by definition, they include any Robinson proteins with extensive β-sheet structures that can bind to membranes mainly by ionic and other interactions^3,5^.

Soon after the F-MMM proposal^1^, it became apparent that another class of membrane proteins was needed. This new class of membrane-associated proteins was proposed, even thought they are not strictly membrane proteins^9^. They are cytoskeletal and associated signaling proteins at the inner membrane surface and certain glycoproteins and linked glycosaminoglycans at the outer membrane surface (see Figure 1 of reference^9^). These membrane-associated components are thought to be involved in stabilizing membranes (and thus cells) and immobilizing membrane components to the extracellular matrix or to cytoskeletal networks inside cells. There they can function as parts of adhesion structures or cell motility traction points. Thus, these protein components are membrane-associated but not directly involved in the integral microstructure of cell membranes, and membrane structure is not dependent on their presence. However, they are important in maintaining membrane function and dynamics, and they are especially important in various cellular activities, such as cell adhesion and its stabilization, cell motility and spreading, endocytosis, exocytosis and many other important actions^9^.

## Cytoskeletal and Extracellular Matrix Membrane interactions

Cytoskeletal and extracellular matrix membrane-associated interactions can alter cell membrane macrostructure by causing reductions or restrictions in freedom of movement or mobility and by causing global movements of membrane glycoprotein and lipid domains by tethering these complexes to cellular or extracellular structures^9,10^. This later situation can occur when cell membrane-associated actin-containing cytoskeletal components are involved in moving or restraining trans-membrane integral membrane proteins through intermediate peripheral membrane proteins and other components^9^.

Even by 1972 there were examples of restriction of mobility of integral membrane components and involvement of the cytoskeleton in translocation of membrane components. For example, during antigen capping certain initially mobile cell surface antigens, even when present in small mobile clusters, were found to be trapped into large, relatively immobile trans-membrane complexes in a temperature- and energy-dependent process involving cytoskeletal elements^65-67^. This process eventually resulted in cytoskeletal-mediated endocytosis of some but not all of the large macromolecular complexes (receptor-rich domains or “receptor patches”)^68^. We now know in lymphoid cells that antigen clustering, domain formation, internalization, acidification of the resulting endosomes, degradation, and membrane recycling are all part of the normal lymphocyte activation process^68^.

The organizational structures that mediate trans-membrane linkages between clusters of integral membrane receptors and the cytoskeleton were ultimately found to be much more complex than the cartoons of the day^9^. They are now thought to involve multiple membrane peripheral proteins, lipid-protein-receptor domains, and enzymes that assemble into a submembrane plaque or supramolecular structure that secures the membrane to a complex system of cytoskeletal elements^69-71^.

The mobility of integral membrane components can also be restricted by cell-cell and cell-matrix interactions. When cells are bound to other cells or to extracellular matrix, at least some of their membrane receptors are immobilized in the process^72-74^.

Cell adhesion complexes that are immobilized by matrix interactions are capable in some cells of communicating signals that are transmitted through a dynamically assembled actin-containing cyto-skeleton to generate mechanical forces that can move cells or resist exterior mechanical stresses^75,76^. This serial system of highly specialized glycoproteins and proteins (extracellular matrix, integral membrane proteins, peripheral membrane proteins, adaptor proteins, cytoskeletal elements, among other components) may have evolved to convert biochemical signals into mechanical forces that are important in cellular behavior. Along with better-known biochemical signaling pathways, these molecular-mechanical pathways have been proposed to be key regulators of cell function^76^.

Early proposals on the role of membrane-associated cytoskeletal elements and their influence on membrane structure and dynamics have proven to be relatively simplistic^9^. Even now this complex system is being carefully dissected and its multiple subcomponents identified^71,77-82^. For example, we now know that certain membrane-interacting components, such as septins, GTPases, and other components, can form higher-order bundles, filaments and even ring structures that bind to actin filaments and microtubules^83-85^. Although membrane peripheral proteins have been identified in cytoskeletal interactions^86^, membrane lipids are also important in such interactions. Indeed, specialized phospholipids may regulate interactions between certain membrane lipid domains, such as lipid rafts (specialized lipid domains), using phosphatidylinositol isoform-binding proteins^87^.

Thus, cells can be considered completely integrated mechanostructures, and cell membranes are not autonomous and separate from other intracellular membranes and organelles. They are continuously interacting with other cellular structures-receiving signals, directing contacts, sending instructions, maintaining cellular polarity and mechanical properties, while undergoing constant turnover of their constituent components.

## Membrane Protein-Protein Interactions

The majority of membrane proteins and glycoproteins are not likely to be isolated compo-nents or complexes floating freely in a fluid lipid environment, as in **Figure 1**^1^. Functionally they are often assembled into macromolecular complexes, for example, to initiate signaling process that are important in a variety of cellular functions. As their cellular and biochemical functions have been elucidated over the years, it has become much clearer how super-complexes of membrane proteins and glycoproteins (domains) perform a variety of cellular functions.

To demonstrate their functional activities membrane proteins have been cloned, sequenced and functionally expressed^15-17^. In such studies, single membrane components can be involved, but usually membrane protein and glycoprotein complexes are not made up of single proteins or glycoproteins^88-90^.

Membrane integral protein-protein interac-tions, which can be driven by ligand binding, are involved in the dynamic formation of trans-membrane signaling complexes. Eventually the complexes can become activated for recruitment of additional peripheral proteins at the inner cell membrane surface to form supramolecular trans-membrane structures that are competent for cellular signaling^76,90-92^. For example, upon binding of cell surface trans-membrane integrin receptors to their ligand, the integrin heterodimers are thought to undergo a ‘bending’ conformational change that allows recruitment of submembrane plaque proteins that, in turn, directly or indirectly bind to actin complexes linked to the cytoskeleton^78^. Subsequently a potentially larger group of other signaling molecules and enzymes can be bound to the submembrane supramolecular complexes, leading to the formation of stable, trans-membrane super-complexes^78,92^.

There are a variety of cell membrane glycoprotein-protein oligomeric complexes that are involved in cell-cell interactions and the formation of specialized super-structures between adjacent cells in tissues. These will not be discussed here, but the reader can find additional examples elsewhere^10^.

## Membrane Lipid-Lipid Interactions

As discussed above, membrane lipids are asymmetrically arranged in cell membranes (reviewed in^20,21,54-56,58,60^). They are also unevenly distributed in the membrane plane (reviewed in^30,44,62,93-96^). Certain lipids change the fluidity, dynamics and lateral structures of cell membranes, such as cholesterol, which as the only sterol present and the single most abundant lipid in animal cell membranes is particularly important in the formation of membrane lipid domains^21,62,95-97^. Lipid-lipid interaction studies using mixtures of membrane phospholipids, cholesterol and sphingomyelin have shown the importance of domain structures in model lipid membranes^62,98,99^.

Cholesterol is particularly important in cell membrane lipid organization^62,96-99^. This is thought to be due, in part, to cholesterol’s “schizophrenic” affinity for fluid and solid phases of membrane lipid bilayers^96^. Cholesterol partitions into liquid ordered and disordered phases to roughly the same extent and changes the properties of different lipid phases or lipid domains^95^.

In plasma membranes sphingolipids are also important in the formation of more-ordered membrane lipid domains^35,94^. Sphingomyelins and phosphatidylcholines constitute more than one-half of plasma membrane phospholipids and form the main partners for cholesterol interactions^94,100^. Sphingomyelins and cholesterol are critically important in the separation and formation of ordered lipid domains (lipid rafts) that are generally surrounded by liquid phase lipids^20,94,101^.

The formation of more ordered lipid phases in plasma membranes is generally important in membrane domain formation and, in particular, the lipid raft hypothesis^20,35,44,45^. The formation of membrane lipid domains or rafts is now thought to be a dynamic and reversible process that confers functional change at the outer surface of plasma membranes^30,35,45,102-104^. Lipid domain formation appears to be driven by multiple forces, such as hydrogen bonding, hydrophobic entropic forces, charge pairing and van der Waals forces^62,101^.

Lipid domains or rafts in plasma membranes contain specific lipids, integral proteins and peripheral proteins, and they can be platforms for signal transduction and other cellular functions^35,104-106^. Plasma membrane lipid rafts are now thought to constitute functional, dynamic nano-sized domains of diameter <300 nm (most ~10-200 nm) that are characterized by enrichments of cholesterol and sphingolipids^31,37,45,107^. However, cell membrane lipid rafts were found to be much smaller than the lipid domains found in artificial membrane bilayers, and their boundary lipids were found to exchange rapidly (every 10-100 nsec) with lipids in the bulk membrane. They also tended to exclude unsaturated phospholipids and cholesterol from their boundaries^108^.

Integral and peripheral membrane proteins may be sequestered into plasma membrane lipid rafts^30,104,106,109^. Neuman et al.^106^ have discussed the properties of lipid rafts (and other membrane domains) that make them biologically important, such as their involvement in cellular processes: endocytosis, signal transduction, cell death, among other events. Lipid rafts are dynamic structures, and the lipids in these domains can quickly exchange with lipids in the bulk fluid membrane as well as other rafts. Neuman et al. speculated that there may be different turnover rates for each raft constituent, and a spectrum of submicro- or nano-domains may exist that contain different lipid and protein compositions, physical characteristics and functions^106^. There may be a limited number of allowed lipid compositions or combinations that can form lipid domains, and these domains are not randomly distributed; they tend to adopt a superlattice structure in membranes^110^.

## Membrane Lipid-Protein interactions

As mentioned in the section above, integral membrane proteins can interact with different membrane domains, but they must also interact with membrane lipids, in order to produce an intact plasma membrane. Specifically, portions of their structures must directly interact with the acyl chain portions of membrane phospholipids or the hydrophobic portions of other membrane lipids. This is accomplished by hydrophobic matching between the hydrophobic lipid bilayer acyl core and a stretch or combination of hydrophobic amino acids^15,16,21,43,48,111^.

The concept of hydrophobic matching between the hydrophobic core of the lipid bilayer and hydrophobic stretches of amino acids in integral membrane proteins was essential for understanding the formation of a stable membrane structure^96,111-114^. If the hydrophobic portions of this structure are mismatched, an elastic distortion of the lipid matrix around the integral membrane protein occurs^48,96,114^. In order to produce an appropriate structure hydrophobic matching of particular lipids adjacent to membrane proteins (boundary lipids) must take place, or there will be an energy penalty that results in an elastic distortion of the lipid matrix immediately around the integral protein^96,111-114^. If the penalty is large enough, the integral protein may undergo a conformational change, and this could potentially cause effects on protein function. It could also affect protein-protein interactions and result in integral protein aggregation in the membrane plane^114^.

There exists a range of protein interactions with lipids in cellular membranes, and these are apparently controlled by the coherence lengths of the interactions between proteins and their boundary membrane lipids. If very large, this could result in capillary-condensation phenomena and wetting around the integral protein^43,115^. Other interactions, such as electrostatic interactions between charged amino acids and phospholipids, can complicate this picture, and Mouritsen^114^ anticipated that under certain circumstances electrostatic interactions might even overcome hydrophobic matching. Lipid preference for certain integral proteins is proposed to result in capillary condensation, and if this occurs around two or more integral membrane proteins, it can result in wetting and the formation of a capillary condensate between adjacent integral proteins. This can produce a lipid-mediated force that drives the formation and stabilization of integral protein complexes^96,114,115^.

The hydrophobic matching principle may also be an essential property in the formation of specialized lipid domains or rafts (see previous section), and it could also be an important mechanism for selective partitioning of integral proteins into specialized membrane lipid domains. To be sequestered into a lipid domain an integral protein’s hydrophobic structure must match up with the hydrophobic thickness of the domain^115^. If the hydrophobic portions of this structure are mismatched, there will be an elastic distortion of the boundary lipid matrix around the integral membrane protein. Without hydrophobic matching of particular boundary lipids immediately adjacent to particular membrane proteins, there will be an energy penalty that causes an elastic distortion of the boundary lipid matrix^96,114^. If the energy penalty is large enough, the integral protein may undergo a conformational change, and this could potentially cause effects on protein function. It can also result in the exclusion of certain lipids, such as cholesterol, from the boundary lipid layer due to unfavorable membrane protein hydrophobic matching^96,114^.

As mentioned above, lipid boundary effects can also cause changes in protein-protein interactions that could result in membrane integral protein aggregation in the membrane plane. By examining the rotational diffusion rates of rhodopsin in reconstituted bilayer membranes, Kusumi and Hyde^108^ were able to relate specific phospholipid acyl chain-lengths to the state of rhodopsin aggregation. They found that hydrophobic mismatch between proteins and lipids is unfavorable energetically, but the mismatching can be minimized by the transient formation of protein-protein complexes.

Another property important in lipid-protein interactions is the tendency of certain lipids to induce curvature stress and the ability of certain membrane peripheral proteins to overcome this stress^53^. This property has some similarities to hydrophobic matching, and the binding of integral proteins to particular lipids could change the conformation of nearby integral proteins, for example, to open or close membrane channels^113^. Alternatively, the binding of peripheral membrane proteins directly to the lipid head groups could decrease or promote lipid curvature as the lipids conform to the protein shape^53^.

In addition to hydrophobic matching of proteins and lipids in cellular membranes, there are additional physical considerations, such as lateral pressure forces, lateral lipid composition and phases, membrane curvature, ionic interactions, among others, that must be taken into account to produce an overall tensionless membrane structure^43,96,112,113,115^.

## Membrane Restrictions on Lateral Mobility

We now know that integral membrane proteins are not necessarily completely free to laterally move in a fluid lipid matrix, as originally proposed^1^. In fact, there are subtle restrictions on the lateral movements of most integral membrane proteins, and at least some lipids, in cellular membranes (reviewed in^9,10,26,27^). Restrictions on the lateral movements of integral membrane proteins have been linked to: (*a*) extracellular restrictions, such as binding to extracellular matrix, (*b*) the formation of specialized membrane domains, such as lipid rafts, (*c*) the formation of large, supramolecular protein complexes and (*d*) the formation of peripheral membrane barriers at the inner membrane surface, such as membrane-associated corrals or skeletal fence works^10,22,23,26-28,41,69^.

**Figure 3:**
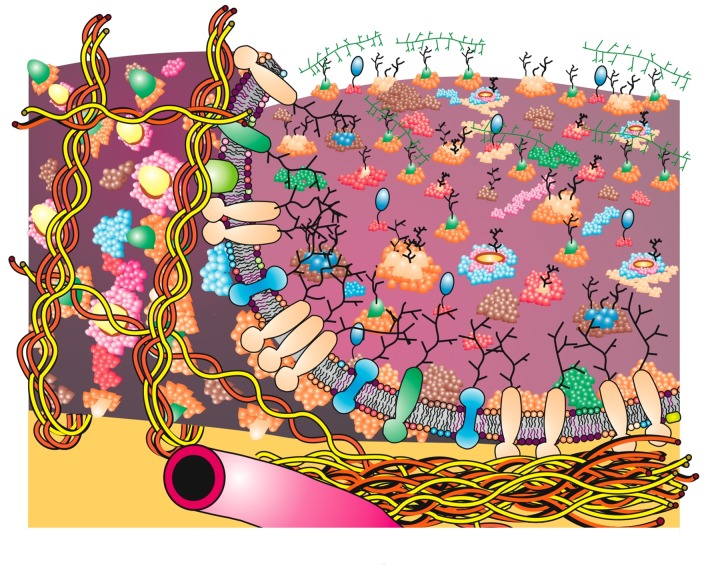
An updated Fluid-Mosaic Membrane Model representation that contains membrane domain structures, membrane - associated cytoskeletal and extracellular structures The cell membrane has been pealed back to the left to reveal the bottom membrane surface and membrane-associated cytoskeletal elements that form barriers (corrals) that limit the lateral motions of some of the integral membrane proteins. In addition, membrane-associated cytoskeletal structures are directly interacting with integral membrane proteins at the inner membrane surface along with matrix components at the outer surface. Although this diagram presents possible mechanisms of integral membrane protein mobility restraint, it does not accurately represent the sizes and structures of integral membrane proteins, lipid domains or membrane-associated cytoskeletal structures.

Reviewing the results from various optical methods that have been used to follow the dynamics of cell surface integral proteins, Jacobson et al.^23^ have placed the lateral movements of membrane proteins into four main categories: (*a*) transient confinement by obstacle protein clusters (fenceposts or pickets) (Figure 2, A); (*b*) transient confinement by the cytoskeletal meshwork into defined domains or corrals (Figure 3, B); (*c*) directed motion by direct or indirect attachment to the cytoskeleton (Figure 5, C); and (*d*) free, random diffusion in the fluid membrane (Figure 5, D). A slightly different list was produced by He and Marguet^35^, who characterized the categories of lateral movement as: (*a*) free diffusion; (*b*) movement limited by meshwork barriers (such as fences or corrals); and (*c*) movement limited by traps and domains (such as lipid rafts). Thus, the original description of integral membrane proteins freely diffusing in the membrane plane (**Figure 1**) pertains to only one of these modes.

It is now well known that a substantial portion of integral membrane proteins are confined, at least transiently, to small membrane domains by lattices and corrals, and they are thus not freely diffusing in the membrane plane (reviewed in^10,26,27^). Integral proteins can escape from one domain to an adjacent domain, and even escape such domains altogether, and this may be related to the sizes of their cytoplasmic structures, their cytoskeletal and extracellular interactions, and their abilities to dyamically undergo protein complex formation^26,27^.

The approximate areas of plasma membrane receptor domains have been estimated from 0.04 to 0.24 μm^2^, and the approximate transit times of membrane receptors in these membrane domains can range from 3 to 30 sec^27-29^. Overall, cell membrane domains can range in diameter from 2-300 nm. For example, actin-cytoskeletal fence domains have been found in the range of 40-300 nm, lipid raft domains in the range of 2-20 nm, and dynamic integral membrane protein complexes in domains of 3-10 nm^27^. Cells must process many types of signal mechanisms, and the use of different types of cell membrane domains may allow another level of signal selection and complexity.

## Membrane Hierarchical Organization

Cells possess dynamic, multi-dimensional plasma membrane architectures so that they can quickly respond to intracellular and extracellular signals, and environmental events. Kusumi and colleagues^26,27^ have proposed that plasma membranes are organized into dynamic hierarchical structures. Within these hierarchical structures membrane components are limited in their diffusion rates from 5- to 50-times slower than when the same components are reconstituted into artificial membranes. Conversely, the macroscopic diffusion rates in cell membranes can also be increased 20-fold through disruption of membrane-associated cytoskeletal networks^27^.

The notion that membrane-associated cytoskeletal networks can slow and limit the mobility of trans-membrane integral proteins compared to free lateral diffusion is not a new concept and was discussed previously^9,22,23,41,116^. Indeed, cytoskeletal-disrupting drugs have been known for some time to change the rates of integral membrane protein diffusion (reviews:^9,66,117^). More recently the impedance of mobility of membrane components can now be directly related in many cell types to cytoskeletal fencing and the formation of cytoskeletal corrals (see **Figure 2**)^23,26-28^.

The partitioning of plasma membranes in order to limit the dynamics of their integral membrane protein components, at least part of the time, to fenced corrals, or tethering them directly or indirectly to membrane-associated cytoskeletal elements, creates relatively stable membrane zones (domains) of enhanced receptor densities. Also, extracellular networks or lattices can potentially partition the plasma membrane into stabilized domains^9,34^. Moreover, the trapping of mobile integral membrane proteins inside a corral constructed of cytoskeletal fencing (or extracellular lattices) may be dependent on the state of protein complex formation. Some integral protein monomers have been found to escape from corrals, but not their oligomeric complexes, or at least they cannot escape at the same rates^28^. Therefore, membrane corrals (or extracellular lattices) may selectively limit free diffusion in the membrane plane, and at the same time they present enhanced receptor densities in specific membrane domains^27^.

Cell membrane dynamic compartmentalization or the enhanced presentation of specific components in specialized domains may be important in signal transduction, cell activation, cell differentiation, identification, and other complex membrane events. This can occur by changing the range of movements, distributions and collision rates of various cellular receptors, and thus affecting their display and ability to associate into higher order complexes. Kusumi et al.^27^ have proposed that plasma membranes possess hierarchical architecture consisting of various membrane domains or compartments, such as: cytoskeletal limited domains or corrals formed by cytoskeletal fences, fenceposts or pickets; lipid raft domains; and dynamic, oligomeric integral membrane protein domains that may or may not be linked to the cytoskeleton, among other possibilities. However, the basic nano-scale membrane unit would still be a fluid-mosaic membrane based on the 1972 model^26,27^.

## An Updated Fluid-Mosaic Membrane Model

Some forty years after the original F-MMM proposal^1^, one would expect that significant and extensive revisions would be necessary^24,25^. However, the basic nano-scale Singer-Nicolson model^1^ has been found worthy, even if it is not entirely accurate when lipid properties, complex membranes domains, and higher hierarchical levels of mosaic organization are considered^10,21,26,27,97^. Although a number of shortcomings of the 1972 model have been noted previously^19,22,27^, the basic nano-scale part of the F-MMM does not require extensive revision beyond the 1976 update published previously^9^. What the F-MMM does require is an update that takes into consideration events and structures above and below the basic membrane, along with some of the unforeseen cell membrane domain and boundary properties absent in the earlier models. Thus an updated F-MMM must take into account contributions that have been made since the 1970s, such as recent data on boundary lipids, membrane lipid and protein domains, cytoskeletal corrals, extracellular matrix, and other properties and conditions that were almost completely unknown in the 1970s. Obviously many of these criticisms cannot be easily addressed in any static cartoon model of the plasma membrane, but most of the important criticisms and newer information have been incorporated into an undated model (**Figure 3**). What is difficult to portray in any cartoon is the concept that the components of cell membranes function in various domains as non-uniform, non-random, cooperative, dynamic elements in thermodynamic equilibrium^21^.

Finally, as discussed throughout this brief review, the plasma membrane is not a static, autonomous cellular structure. It is linked in several ways to the cell cytoplasm through cytoskeletal networks, signal transduction systems, transport systems, and other structural, enzymatic and communication networks. In tissues, it is also linked outside the cell to extracellular matrix, other cells, and to interstitial protein structures. Thus, cell membranes are fully integrated structures within tissues, and they are sensitive and reactive to environmental changes and signals. This is probably why plasma membranes have evolved to become such complex, dynamic structures. They have to quickly and selectively respond to a number of disparate signals from within and outside cells. These subtle, dynamic and sensitive cellular structures will continue to fascinate researchers who seek to uncover their secretes.

## Key Point

****An updated Fluid-Mosaic Membrane Model****, incorporating **membrane domains and restraints on the mobility**/range of motion of membrane components.

## References

[1] Singer S J, Nicolson G L (1972). The fluid mosaic model of the structure of cell membranes.. Science (New York, N.Y.).

[2] Danielli James Frederic, Davson Hugh (1935). A contribution to the theory of permeability of thin films. Journal of Cellular and Comparative Physiology.

[3] ROBERTSON J D (1959). The ultrastructure of cell membranes and their derivatives.. Biochemical Society symposium.

[4] Gorter E, Grendel F (1925). ON BIMOLECULAR LAYERS OF LIPOIDS ON THE CHROMOCYTES OF THE BLOOD.. The Journal of experimental medicine.

[5] ROBERTSON J D (1960). The molecular structure and contact relationships of cell membranes.. Progress in biophysics and molecular biology.

[6] Pinto da Silva P, Branton D (1970). Membrane splitting in freeze-ethching. Covalently bound ferritin as a membrane marker.. The Journal of cell biology.

[7] Benson A A (1966). On the orientation of lipids in chloroplast and cell membranes.. Journal of the American Oil Chemists' Society.

[8] Green D E, Allmann D W, Bachmann E, Baum H, Kopaczyk K, Korman E F, Lipton S, MacLennan D H, McConnell D G, Perdue J F, Rieske J S, Tzagoloff A (1967). Formation of membranes by repeating units.. Archives of biochemistry and biophysics.

[9] Nicolson G L (1976). Transmembrane control of the receptors on normal and tumor cells. I. Cytoplasmic influence over surface components.. Biochimica et biophysica acta.

[10] Nicolson Garth L (2014). The Fluid-Mosaic Model of Membrane Structure: still relevant to understanding the structure, function and dynamics of biological membranes after more than 40 years.. Biochimica et biophysica acta.

[11] NICOLSON Garth L., POSTE George, JI Tae H. (1977). The dynamics of cell membrane organization. Dynamic Aspects of Cell Surface Organization.

[12] Edidin Michael (2003). Timeline: Lipids on the frontier: a century of cell-membrane bilayers. Nature Reviews Molecular Cell Biology.

[13] SINGER S.J. (1971). THE MOLECULAR ORGANIZATION OF BIOLOGICAL MEMBRANES. Structure and Function of Biological Membranes.

[14] Singer S J (1974). The Molecular Organization of Membranes. Annual Review of Biochemistry.

[15] Singer S. J. (1990). The Structure and Insertion of Integral Proteins in Membranes. Annual Review of Cell Biology.

[16] von Heijne Gunnar (1988). Transcending the impenetrable: How proteins come to terms with membranes. Biochimica et Biophysica Acta (BBA) - Reviews on Biomembranes.

[17] von Heijne Gunnar (2006). Membrane-protein topology. Nature Reviews Molecular Cell Biology.

[18] Bretscher M. S. (1973). Membrane Structure: Some General Principles. Science.

[19] De Weer Paul (2000). A Century of Thinking About Cell Membranes. Annual Review of Physiology.

[20] van Meer Gerrit, Voelker Dennis R, Feigenson Gerald W (2008). Membrane lipids: where they are and how they behave.. Nature reviews. Molecular cell biology.

[21] Bagatolli Luis A, Ipsen John H, Simonsen Adam C, Mouritsen Ole G (2010). An outlook on organization of lipids in membranes: searching for a realistic connection with the organization of biological membranes.. Progress in lipid research.

[22] Zhang F, Lee G M, Jacobson K (1993). Protein lateral mobility as a reflection of membrane microstructure.. BioEssays : news and reviews in molecular, cellular and developmental biology.

[23] Jacobson K, Sheets E D, Simson R (1995). Revisiting the fluid mosaic model of membranes.. Science (New York, N.Y.).

[24] Vereb G, Szöllosi J, Matkó J, Nagy P, Farkas T, Vigh L, Mátyus L, Waldmann T A, Damjanovich S (2003). Dynamic, yet structured: The cell membrane three decades after the Singer-Nicolson model.. Proceedings of the National Academy of Sciences of the United States of America.

[25] Wiśniewska Anna, Draus Jolanta, Subczynski Witold K (2003). Is a fluid-mosaic model of biological membranes fully relevant? Studies on lipid organization in model and biological membranes.. Cellular & molecular biology letters.

[26] Kusumi Akihiro, Suzuki Kenichi G N, Kasai Rinshi S, Ritchie Ken, Fujiwara Takahiro K (2011). Hierarchical mesoscale domain organization of the plasma membrane.. Trends in biochemical sciences.

[27] Kusumi Akihiro, Fujiwara Takahiro K., Chadda Rahul, Xie Min, Tsunoyama Taka A., Kalay Ziya, Kasai Rinshi S., Suzuki Kenichi G.N. (2012). Dynamic Organizing Principles of the Plasma Membrane that Regulate Signal Transduction: Commemorating the Fortieth Anniversary of Singer and Nicolson's Fluid-Mosaic Model. Annual Review of Cell and Developmental Biology.

[28] Kusumi Akihiro, Sako Yasushi (1996). Cell surface organization by the membrane skeleton. Current Opinion in Cell Biology.

[29] Kusumi A, Sako Y, Yamamoto M (1993). Confined lateral diffusion of membrane receptors as studied by single particle tracking (nanovid microscopy). Effects of calcium-induced differentiation in cultured epithelial cells.. Biophysical journal.

[30] Jacobson Ken, Mouritsen Ole G, Anderson Richard G W (2007). Lipid rafts: at a crossroad between cell biology and physics.. Nature cell biology.

[31] Simons Kai, Toomre Derek (2000). Lipid rafts and signal transduction. Nature Reviews Molecular Cell Biology.

[32] Lavi Yael, Edidin Michael A, Gheber Levi A (2007). Dynamic patches of membrane proteins.. Biophysical journal.

[33] Escribá Pablo V., González-Ros José M., Goñi Félix M., Kinnunen Paavo K. J., Vigh Lászlo, Sánchez-Magraner Lissete, Fernández Asia M., Busquets Xavier, Horváth Ibolya, Barceló-Coblijn Gwendolyn (2008). Membranes: a meeting point for lipids, proteins and therapies. Journal of Cellular and Molecular Medicine.

[34] Lajoie Patrick, Goetz Jacky G., Dennis James W., Nabi Ivan R. (2009). Lattices, rafts, and scaffolds: domain regulation of receptor signaling at the plasma membrane. The Journal of Cell Biology.

[35] Lingwood D., Simons K. (2009). Lipid Rafts As a Membrane-Organizing Principle. Science.

[36] He Hai-Tao, Marguet Didier (2011). Detecting Nanodomains in Living Cell Membrane by Fluorescence Correlation Spectroscopy. Annual Review of Physical Chemistry.

[37] Quinn Peter J. (2010). A lipid matrix model of membrane raft structure. Progress in Lipid Research.

[38] Kauzmann W. (1959). Some Factors in the Interpretation of Protein Denaturation. Advances in Protein Chemistry.

[39] Cramer W A, Engelman D M, Von Heijne G, Rees D C (1992). Forces involved in the assembly and stabilization of membrane proteins.. FASEB journal : official publication of the Federation of American Societies for Experimental Biology.

[40] Israelachvili J N (1977). Refinement of the fluid-mosaic model of membrane structure.. Biochimica et biophysica acta.

[41] Jacobson K, Ishihara A, Inman R (1987). Lateral diffusion of proteins in membranes.. Annual review of physiology.

[42] Watts A. (1989). Membrane structure and dynamics. Current Opinion in Cell Biology.

[43] Gil T, Ipsen J H, Mouritsen O G, Sabra M C, Sperotto M M, Zuckermann M J (1998). Theoretical analysis of protein organization in lipid membranes.. Biochimica et biophysica acta.

[44] Simons Kai, Vaz Winchil L C (2004). Model systems, lipid rafts, and cell membranes.. Annual review of biophysics and biomolecular structure.

[45] Simons Kai, Sampaio Julio L (2011). Membrane organization and lipid rafts.. Cold Spring Harbor perspectives in biology.

[46] Zimmerberg Joshua, Gawrisch Klaus (2006). The physical chemistry of biological membranes.. Nature chemical biology.

[47] McMahon Harvey T., Gallop Jennifer L. (2005). Membrane curvature and mechanisms of dynamic cell membrane remodelling. Nature.

[48] Mouritsen O.G., Bloom M. (1984). Mattress model of lipid-protein interactions in membranes. Biophysical Journal.

[49] Chernomordik Leonid V., Kozlov Michael M. (2003). Protein-Lipid Interplay in Fusion and Fission of Biological Membranes. Annual Review of Biochemistry.

[50] Zimmerberg Joshua, Kozlov Michael M (2006). How proteins produce cellular membrane curvature.. Nature reviews. Molecular cell biology.

[51] Baumgart Tobias, Capraro Benjamin R., Zhu Chen, Das Sovan L. (2011). Thermodynamics and Mechanics of Membrane Curvature Generation and Sensing by Proteins and Lipids. Annual Review of Physical Chemistry.

[52] Frost Adam, Unger Vinzenz M., De Camilli Pietro (2009). The BAR Domain Superfamily: Membrane-Molding Macromolecules. Cell.

[53] Antonny Bruno (2006). Membrane deformation by protein coats. Current Opinion in Cell Biology.

[54] Rothman J., Lenard J (1977). Membrane asymmetry. Science.

[55] Etemadi Abol-Hassan (1980). Membrane asymmetry A survey and critical appraisal of the methodology II. Methods for assessing the unequal distribution of lipids. Biochimica et Biophysica Acta (BBA) - Biomembranes.

[56] Stoeckenius W. (1969). CURRENT MODELS FOR THE STRUCTURE OF BIOLOGICAL MEMBRANES. The Journal of Cell Biology.

[57] Pomorski Thomas, Hrafnsdóttir Sigrún, Devaux Philippe F., Meer Gerrit van (2001). Lipid distribution and transport across cellular membranes. Seminars in Cell & Developmental Biology.

[58] Daleke David L. (2002). Regulation of transbilayer plasma membrane phospholipid asymmetry. Journal of Lipid Research.

[59] Sharom Frances J. (2011). Flipping and flopping-lipids on the move. IUBMB Life.

[60] Quinn Peter J. (2002). Plasma Membrane Phospholipid Asymmetry. Subcellular Biochemistry.

[61] Brown D.A., London E. (1998). Structure and Origin of Ordered Lipid Domains in Biological Membranes. Journal of Membrane Biology.

[62] Ohvo-Rekilä Henna, Ramstedt Bodil, Leppimäki Petra, Slotte J Peter (2002). Cholesterol interactions with phospholipids in membranes.. Progress in lipid research.

[63] Silvius John R (2005). Partitioning of membrane molecules between raft and non-raft domains: insights from model-membrane studies.. Biochimica et biophysica acta.

[64] von Heijne G (1999). Recent advances in the understanding of membrane protein assembly and structure.. Quarterly reviews of biophysics.

[65] Edidin M, Weiss A (1972). Antigen cap formation in cultured fibroblasts: a reflection of membrane fluidity and of cell motility.. Proceedings of the National Academy of Sciences of the United States of America.

[66] Poste G, Papahadjopoulos D, Nicolson G L (1975). Local anesthetics affect transmembrane cytoskeletal control of mobility and distribution of cell surface receptors.. Proceedings of the National Academy of Sciences of the United States of America.

[67] Bourguignon L Y, Singer S J (1977). Transmembrane interactions and the mechanism of capping of surface receptors by their specific ligands.. Proceedings of the National Academy of Sciences of the United States of America.

[68] Unanue E. R. (1972). LIGAND-INDUCED MOVEMENT OF LYMPHOCYTE MEMBRANE MACROMOLECULES: I. ANALYSIS BY IMMUNOFLUORESCENCE AND ULTRASTRUCTURAL RADIOAUTOGRAPHY. Journal of Experimental Medicine.

[69] Geiger B, Yehuda-Levenberg S, Bershadsky A D (1995). Molecular interactions in the submembrane plaque of cell-cell and cell-matrix adhesions.. Acta anatomica.

[70] Fuchs E, Cleveland D W (1998). A structural scaffolding of intermediate filaments in health and disease.. Science (New York, N.Y.).

[71] Chichili Gurunadh R, Rodgers William (2009). Cytoskeleton-membrane interactions in membrane raft structure.. Cellular and molecular life sciences : CMLS.

[72] Nicolson G L, Poste G (1979). Lectin-mediated agglutination of murine lymphoma cells. Cell surface deformability and reversibility of agglutination by saccharides.. Biochimica et biophysica acta.

[73] Salas P J, Vega-Salas D E, Hochman J, Rodriguez-Boulan E, Edidin M (1988). Selective anchoring in the specific plasma membrane domain: a role in epithelial cell polarity.. The Journal of cell biology.

[74] Kobialka Szymon, Beuret Nicole, Ben-Tekaya Houchaima, Spiess Martin (2009). Glycosaminoglycan chains affect exocytic and endocytic protein traffic.. Traffic (Copenhagen, Denmark).

[75] Geiger Benjamin (1983). Membrane-cytoskeleton interaction. Biochimica et Biophysica Acta (BBA) - Reviews on Biomembranes.

[76] Roca-Cusachs Pere, Iskratsch Thomas, Sheetz Michael P (2012). Finding the weakest link: exploring integrin-mediated mechanical molecular pathways.. Journal of cell science.

[77] Geiger B, Bershadsky A (2001). Assembly and mechanosensory function of focal contacts.. Current opinion in cell biology.

[78] Geiger B, Bershadsky A, Pankov R, Yamada K M (2001). Transmembrane crosstalk between the extracellular matrix--cytoskeleton crosstalk.. Nature reviews. Molecular cell biology.

[79] Sheetz M P (2001). Cell control by membrane-cytoskeleton adhesion.. Nature reviews. Molecular cell biology.

[80] Parsons J Thomas, Horwitz Alan Rick, Schwartz Martin A (2010). Cell adhesion: integrating cytoskeletal dynamics and cellular tension.. Nature reviews. Molecular cell biology.

[81] Anitei Mihaela, Hoflack Bernard (2011). Bridging membrane and cytoskeleton dynamics in the secretory and endocytic pathways.. Nature cell biology.

[82] Jaqaman Khuloud, Grinstein Sergio (2012). Regulation from within: the cytoskeleton in transmembrane signaling.. Trends in cell biology.

[83] Weirich Christine S, Erzberger Jan P, Barral Yves (2008). The septin family of GTPases: architecture and dynamics.. Nature reviews. Molecular cell biology.

[84] Hagiwara Akari, Tanaka Yasuhiro, Hikawa Rie, Morone Nobuhiro, Kusumi Akihiro, Kimura Hiroshi, Kinoshita Makoto (2011). Submembranous septins as relatively stable components of actin-based membrane skeleton.. Cytoskeleton (Hoboken, N.J.).

[85] Mostowy Serge, Cossart Pascale (2012). Septins: the fourth component of the cytoskeleton.. Nature reviews. Molecular cell biology.

[86] Schwarz Ulrich S, Gardel Margaret L (2012). United we stand: integrating the actin cytoskeleton and cell-matrix adhesions in cellular mechanotransduction.. Journal of cell science.

[87] Janmey Paul A, Lindberg Uno (2004). Cytoskeletal regulation: rich in lipids.. Nature reviews. Molecular cell biology.

[88] Hollenberg M D (1991). Structure-activity relationships for transmembrane signaling: the receptor's turn.. FASEB journal : official publication of the Federation of American Societies for Experimental Biology.

[89] Jordan J D, Landau E M, Iyengar R (2000). Signaling networks: the origins of cellular multitasking.. Cell.

[90] Cho Wonhwa (2006). Building signaling complexes at the membrane.. Science's STKE : signal transduction knowledge environment.

[91] Damjanovich S, Gáspár R, Pieri C (1997). Dynamic receptor superstructures at the plasma membrane.. Quarterly reviews of biophysics.

[92] Campbell Iain D, Humphries Martin J (2011). Integrin structure, activation, and interactions.. Cold Spring Harbor perspectives in biology.

[93] Somerharju Pentti, Virtanen Jorma A., Cheng Kwan Hon (1999). Lateral organisation of membrane lipids. Biochimica et Biophysica Acta (BBA) - Molecular and Cell Biology of Lipids.

[94] Ramstedt Bodil, Slotte J Peter (2006). Sphingolipids and the formation of sterol-enriched ordered membrane domains.. Biochimica et biophysica acta.

[95] Lindblom Göran, Orädd Greger (2009). Lipid lateral diffusion and membrane heterogeneity.. Biochimica et biophysica acta.

[96] Mouritsen Ole G (2011). Model answers to lipid membrane questions.. Cold Spring Harbor perspectives in biology.

[97] Quinn Peter J (2012). Lipid-lipid interactions in bilayer membranes: married couples and casual liaisons.. Progress in lipid research.

[98] Aittoniemi Jussi, Niemelä Perttu S, Hyvönen Marja T, Karttunen Mikko, Vattulainen Ilpo (2007). Insight into the putative specific interactions between cholesterol, sphingomyelin, and palmitoyl-oleoyl phosphatidylcholine.. Biophysical journal.

[99] Zhang Zhancheng, Bhide Shreyas Y, Berkowitz Max L (2007). Molecular dynamics simulations of bilayers containing mixtures of sphingomyelin with cholesterol and phosphatidylcholine with cholesterol.. The journal of physical chemistry. B.

[100] Lingwood Daniel, Kaiser Hermann-Josef, Levental Ilya, Simons Kai (2009). Lipid rafts as functional heterogeneity in cell membranes.. Biochemical Society transactions.

[101] Quinn Peter J, Wolf Claude (2009). The liquid-ordered phase in membranes.. Biochimica et biophysica acta.

[102] Simons K, Ikonen E (1997). Functional rafts in cell membranes.. Nature.

[103] Mayor Satyajit, Rao Madan (2004). Rafts: scale-dependent, active lipid organization at the cell surface.. Traffic (Copenhagen, Denmark).

[104] Simons Kai, Gerl Mathias J (2010). Revitalizing membrane rafts: new tools and insights.. Nature reviews. Molecular cell biology.

[105] Sengupta Prabuddha, Baird Barbara, Holowka David (2007). Lipid rafts, fluid/fluid phase separation, and their relevance to plasma membrane structure and function.. Seminars in cell & developmental biology.

[106] Neumann Aaron K, Itano Michelle S, Jacobson Ken (2010). Understanding lipid rafts and other related membrane domains.. F1000 biology reports.

[107] Suzuki Kenichi G N (2012). Lipid rafts generate digital-like signal transduction in cell plasma membranes.. Biotechnology journal.

[108] Kusumi A, Hyde J S (1982). Spin-label saturation-transfer electron spin resonance detection of transient association of rhodopsin in reconstituted membranes.. Biochemistry.

[109] Sharma Pranav, Varma Rajat, Sarasij R C, Ira, Gousset Karine, Krishnamoorthy G, Rao Madan, Mayor Satyajit (2004). Nanoscale organization of multiple GPI-anchored proteins in living cell membranes.. Cell.

[110] Somerharju Pentti, Virtanen Jorma A, Cheng Kwan H, Hermansson Martin (2009). The superlattice model of lateral organization of membranes and its implications on membrane lipid homeostasis.. Biochimica et biophysica acta.

[111] Israelachvili J. N., Marčelja S., Horn R. G. (1980). Physical principles of membrane organization. Quarterly Reviews of Biophysics.

[112] Dumas Fabrice, Lebrun Maria Chantal, Tocanne Jean-François (1999). Is the protein/lipid hydrophobic matching principle relevant to membrane organization and functions?. FEBS Letters.

[113] Andersen Olaf S., Koeppe Roger E. (2007). Bilayer Thickness and Membrane Protein Function: An Energetic Perspective. Annual Review of Biophysics and Biomolecular Structure.

[114] Mouritsen Ole G. (2011). Lipids, curvature, and nano-medicine. European Journal of Lipid Science and Technology.

[115] Gil T, Sabra M C, Ipsen J H, Mouritsen O G (1997). Wetting and capillary condensation as means of protein organization in membranes.. Biophysical journal.

[116] Edidin M, Kuo S C, Sheetz M P (1991). Lateral movements of membrane glycoproteins restricted by dynamic cytoplasmic barriers.. Science (New York, N.Y.).

[117] Doherty Gary J., McMahon Harvey T. (2008). Mediation, Modulation, and Consequences of Membrane-Cytoskeleton Interactions. Annual Review of Biophysics.

